# Advanced Ultrasound and Photoacoustic Imaging in Cardiology

**DOI:** 10.3390/s21237947

**Published:** 2021-11-28

**Authors:** Min Wu, Navchetan Awasthi, Nastaran Mohammadian Rad, Josien P. W. Pluim, Richard G. P. Lopata

**Affiliations:** 1 Photoacoustics and Ultrasound Laboratory Eindhoven (PULS/e), Department of Biomedical Engineering, Eindhoven University of Technology, 5612 AZ Eindhoven, The Netherlands; n.mohammadian.rad@tue.nl (N.M.R.); r.lopata@tue.nl (R.G.P.L.); 2 Medical Image Analysis Group (IMAG/e), Department of Biomedical Engineering, Eindhoven University of Technology, 5612 AZ Eindhoven, The Netherlands; j.pluim@tue.nl

**Keywords:** cardiovascular diseases, ultrasound imaging, photoacoustic imaging, segmentation, deep learning, vulnerable plaques

## Abstract

Cardiovascular diseases (CVDs) remain the leading cause of death worldwide. An effective management and treatment of CVDs highly relies on accurate diagnosis of the disease. As the most common imaging technique for clinical diagnosis of the CVDs, US imaging has been intensively explored. Especially with the introduction of deep learning (DL) techniques, US imaging has advanced tremendously in recent years. Photoacoustic imaging (PAI) is one of the most promising new imaging methods in addition to the existing clinical imaging methods. It can characterize different tissue compositions based on optical absorption contrast and thus can assess the functionality of the tissue. This paper reviews some major technological developments in both US (combined with deep learning techniques) and PA imaging in the application of diagnosis of CVDs.

## 1. Introduction

Cardiovascular diseases (CVDs) are a class of diseases affecting the heart and/or the blood vessels. It is still an alarming threat to global health and is responsible for about one third of all deaths, being the number-one killer worldwide [1]. In addition, CVDs is also the major economic burden to the social health-care system due to the substantial direct and indirect cost related to the management of CVDs [2]. For an effective management and treatment of CVDs, accurate diagnosis of the disease and real-time interventional guidance is critical. Various imaging techniques such as X-ray-based imaging (cardiac CT, coronary angiogram), magnetic resonance imaging (MRI) and ultrasound (US) imaging are currently commonly applied in clinics for the diagnosis of CVDs [3]. However, X-ray-based imaging involves a high radiation dose, and MRI is relatively expensive and not always available for frequent, daily use. US imaging is safe, easy to operate, and is known for its high spatial and temporal resolution, low cost, and high accessibility. Therefore, US imaging has become the most commonly used diagnostic imaging technique in cardiology [4].

New imaging techniques are being investigated and developed. Photoacoustic (PA) oroptoacoustic) imaging is a novel imaging technique, taking advantage of both light and sound. In PA imaging, short pulses of laser light are transmitted to irradiate the tissue, and are absorbed in the tissue, generating ultrasound signals due to the thermo-elastic expansion. These ultrasound signals can be received by a conventional US transducer to reconstruct PA images [5]. Generally, the amplitude of the PA signal is proportional to the optical absorption of the tissue. By operating at different optical spectral ranges, the multispectral photoacoustic imaging can reveal the unique wavelength dependent behavior of different materials [6] and is useful to characterize different tissue compositions and assess tissue functionality [7,8,9]. Over recent decades, substantial improvements have been achieved in the field of PA imaging in the diagnosis of CVDs.

As mentioned above, US imaging has been and will remain one of the most widely applied imaging techniques in cardiology in the coming future. PA imaging is intrinsically bonded to and is complementary to US imaging, making it a promising new imaging technique towards clinical applications in cardiology. Furthermore, with the increase in GPU power, deep learning (DL) techniques have gained popularity. DL algorithms require less knowledge about the domain and can capture data features on their own, and hence can be easily applied in complex scenarios [10] while requiring few experts for manual annotations after the model development is complete [11,12]. DL techniques substantially impact the advancement of modern US and PA imaging processing methods. DL techniques generally have become the state-of-the-art methods for segmentation [13,14,15], classification [16], reconstruction [17,18], and registration tasks.

In this paper, we summarized the development of US and PA imaging and the application of DL techniques in both imaging modalities in cardiology. In Section 2, we will first give a condensed overview of the major developments in US imaging and then focus on the DL-based advanced US imaging processing methods. In Section 3, we will first comprehensively review the recent technical advances in PA imaging and then briefly discuss the application of DL-based PA imaging techniques in cardiology. Finally, findings are summarized, and some remaining/future challenges are discussed in Section 4.

## 2. Advanced US Imaging in Cardiology and DL Techniques

The use of ultrasound in cardiology was first introduced by Edler and Hertz [19,20]. They were the first one to record the echoes from the anterior leaflet of the mitral valve. The basic US imaging principle can be found in [21]. Since then, US imaging has evolved to 1-D A and M-mode imaging, real-time 2-D and 3-D B-mode, intravascular US imaging to directly visualize the artery wall from inside, e.g., in the coronaries, and the ultrafast US imaging to better characterize the cardiac functions [22,23,24]. Moreover, US is known for its many functional imaging modalities [4], such as US-based Doppler imaging to measure blood [25], strain imaging to quantify myocardial dynamics [26], shear wave elastography [27], and the use of contrast agents to further improve US imaging quality and flow imaging, and quantify tissue perfusion [28,29].

### 2.1. DL Techniques in US Imaging in Cardiology

**Figure 1:**
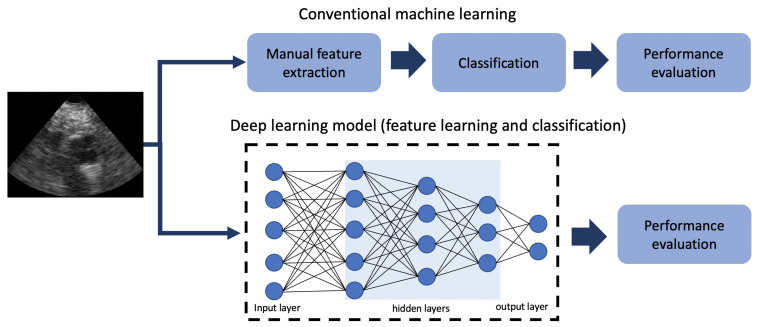
Conventional machine learning vs. DL for a classification task.

Besides the developments in US imaging itself, with the introduction of DL, advanced imaging processing techniques are available and can further improve diagnosis and treatment of CVDs patients [30]. Unlike conventional machine learning algorithms, which mainly rely on manual feature extraction (see Figure 1), DL techniques do not require substantial domain knowledge [31]. Instead, they automatically learn a high-level representation of data.

Advances in DL extend the application of artificial neural network (NN) theory by providing the possibility of training a NN architecture with multiple hidden layers using a backpropagation algorithm [32]. Convolutional neural networks (CNN) [33], recurrent neural networks (RNN) [11], and generative adversarial neural networks (GAN) [34] are the most commonly used deep neural networks (DNN) for cardiovascular image analysis. In the following section, we will selectively focus on reviewing some typical work about the application of diverse DL methods that are gaining increased attention in the field, such as viewpoint classification, Left ventricle segmentation, and intravascular ultrasound segmentation. Furthermore, we state the importance of point of care ultrasound imaging.

#### 2.1.1. Advanced Techniques for Cardiac Viewpoint Classification

Different views of the heart are acquired using a transthoracic echocardiogram (TTE) which can help in understanding the complex anatomy and functions of the heart. These views consist of various video clips, Doppler images from different angles, as well as still images. The information is presented in terms of m-mode recordings, continuous and pulsed wave Doppler imaging. The determination of the view is a very important step in understanding the echocardiogram [35]. This step is challenging as the views sometimes differ very slightly from one another and cannot be classified so easily. The methods generally are time-consuming and require manual intervention by the operator for annotating the features.

**Figure 2:**
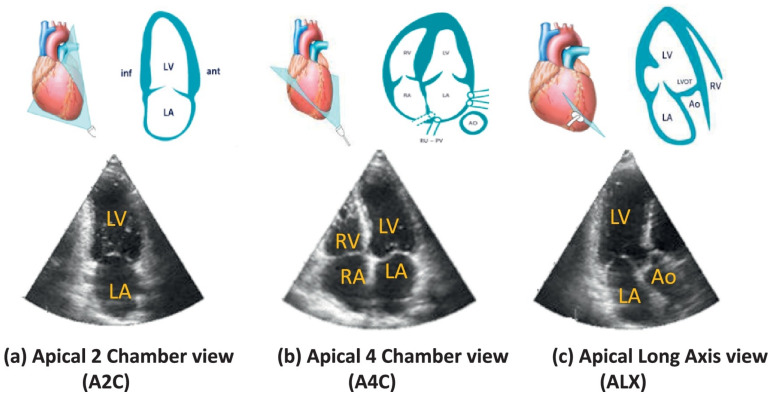
Echocardiographic apical views: (**a**) Apical 2 Chamber view (A2C), (**b**) Apical 4 Chamber view (A4C) and (**c**) Apical Long-Axis view (ALX). (Courtesy and copyrights: 123sonography.com) (Reprinted from [44] with permission).

Various techniques, classical as well as machine learning-based, have been used for classification of echo videos and images. Support vector machines (SVM) and linear discriminant analysis (LDA) have been used as one of the primary tools for classification by learning the decision boundaries and classifying the different views in space [36,37,38,39,40,41]. Multi-class logit-boost classifiers are also proposed for classification of the view in echocardiographic images [42,43]. Khamis et al. [44] proposed a multi-stage classification algorithm for employing spatio-temporal feature extraction and supervised dictionary learning to classify longitudinal scans namely: apical two-chamber (A2C), apical four-chamber (A4C) and apical long-axis (ALX), as shown in Figure 2. The inherent noise makes the classification challenging. Introducing discriminative dictionary learning helped reaching an average accuracy rate of 95% ( 97%, 91% and 97% of A2C, A4C and ALX respectively). Park et al. [45] proposed a probabilistic boosting network principle using the local structure dependence for identifying the cardiac view of B-mode images and then builds on this for inferring the final Doppler gate location in B-mode echocardiograms.

The classical methods for classifying view in echocardiograms are time-consuming and require operator-dependent manual intervention to obtain the desired results. Hence, there has been a wide interest in DL-based approaches for classifying the view of the heart. Penatti et al. [46] proposed a bag of visual words (BOVW) representation for the classification of four cardiac view planes. A BOVW for an image represents an image as a set of features which consists of keypoints and descriptors. Keypoints are the distinct points in the image while the descriptors are the descriptions for the keypoint. The keypoints and the descriptors are used to construct vocabularies of the image and represent the image as a frequency histogram of features. From the frequency histogram, we can predict the category of the image [47]. The technique was robust to noise filtering, down-sampling, and achieved a classification accuracy of 90%. Gao et al. [48] proposed a fused DL-based architecture for integration of spatial as well as temporal information for classifying the echocardiographic videos for eight viewpoints, and achieved an accuracy of 92.1%. Madani et al. [49] proposed a DL-based classification of echocardiograms using CNNs for classifying 15 standard views (3 still and 12 videos) from a large dataset consisting of 267 transthoracic echocardiograms. The model was able to achieve an accuracy of 97.8% and 91.7% for low-resolution images. Another area of research is developing lightweight models for performing viewpoint classification which have fewer parameters and can be used for fast mobile applications for point of care ultrasound applications. Vaseli et al. [50] proposed a lightweight model and used only 1% of the parameters normally comprising a DL model, and achieved a comparable accuracy of 88.1% for 12 view classification in a dataset of 16,612 echograms obtained from 3151 patients.

#### 2.1.2. Advanced Techniques in US Imaging to Improve Left Ventricle Segmentation

Segmentation of the left ventricle (LV) of the heart is a very important step in diagnosing cardiopathies. Segmentation in US echocardiography image sequences is generally challenging, mainly due to the existence of speckle-noise, shadowing, artifacts, and edge dropouts. Earlier studies on cardiac image segmentation rely on deformable models [51], active contours [52], and classical feature extraction techniques [53]. Despite their popularity, these techniques suffer from some limitations. For example, active contours and deformable models need prior knowledge about the tissue shape and appearance [54,55]. Manual feature extraction is a computationally intensive process [56]. Furthermore, it is mainly based on generic researchers’ domain knowledge rather than encoding information in data. Thus, some important information present in the data may be left unused in the segmentation phase.

To tackle the issues mentioned above, recently, DL has been used in cardiac image segmentation and has shown considerable improvement in terms of accuracy and speed [57]. CNN-based models, i.e., fully convolutional neural networks (FCN) [58], U-net [14] and its variations are among the most commonly used DL-based models for cardiac image segmentation. These models have been widely employed for LV segmentation on 2D or 3-D US cardiac images [59,60,61,62,63,64].

The performance of LV segmentation relying on a single DL model might be limited due to the inherent challenges of US images, such as low signal-to-noise ratio, the existence of speckle and resulting low image contrast [65]. To overcome the above limitations and further improve the LV segmentation, several studies have proposed hybrid methods, combining a DL-based segmentation model, such as a CNN, with (i) a classical segmentation model, e.g., a deformable model [66]; or (ii) another DL architecture such as an RNN [67].

In the hybrid framework combining DL-based segmentation and deformable models [65,68,69,70,71], the deformable models act as a post-processing step to refine the output of segmentation. Experimental results of such a hybrid framework in [71] demonstrated the effectiveness of the proposed method in providing accurate segmentation of LV.

Another hybrid framework based on the combination of DL-based segmentation with RNNs was proposed to include spatio-temporal information of data in the learning procedure. In [67], the spatio-temporal information from echocardiography was simultaneously captured by this hybrid framework while segmenting LV structure. The proposed method was applied on the raw echocardiography frames, resulting in a segmentation accuracy of 97.9%.

Elsewhere, Oktay et al. [72] introduced an anatomically constrained CNN for LV segmentation. This model included prior knowledge about the organ’s shape in a CNN through a regularization model, which is based on an autoencoder network. This regularization model encourages the segmentation model to follow the anatomical priors of the underlying anatomy via learned nonlinear representations of the shape. The performance of the proposed segmentation method was evaluated using a Dice score which is defined as a ratio of overlap between the ground truth and the segmentation output, ranging from 0 (no overlap) to 1 (complete overlap). The experimental results on the CETUS’14 challenge dataset [73] showed a high performance with a Dice score of 0.91 for end-diastole and 0.87 for end-systole.

**Figure 3:**
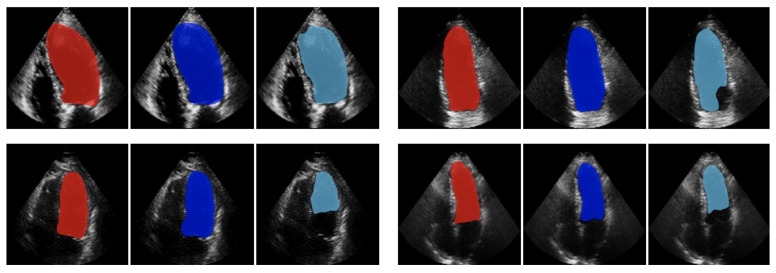
Illustration of LV segmentation for four sample subjects. The results of the semi-supervised method and U-net are shown by blue and cyan colors, respectively. The red color indicates the ground truth. Reprint from [77] with permission.

Most DL architectures applied for LV segmentation are trained in a supervised manner. In supervised learning, data with corresponding labels are given to a network for segmentation or classification purposes. However, data labeling is an expensive and time-consuming task. To overcome these challenges, semi-supervised learning algorithms are used to leverage the unlabeled data for improving the overall performance of LV segmentation [55,74,75]. In a more recent work by Ta et al. [75], a semi-supervised joint learning method was used for simultaneous LV segmentation and motion tracking in 2D+t echocardiographic sequences. A network with two branches, one for motion tracking and another for segmentation tasks, are trained simultaneously such that each branch gradually refines the result of the other. Their proposed method for LV segmentation showed the Dice score of 0.95±0.01 on synthetic human echocardiographic sequences and 0.87±0.01 on in vivo canine models. This framework was also applied on 3D+t echocardiographic sequences to further improve the segmentation and motion tracking of LV [76]. Jafari et al. [77] presented a semi-supervised learning framework based on a hybrid DL model comprised of a generative model and U-net for LV segmentation. The model was trained on the whole cine where the ground truth was only available for end-diastolic and end-systolic frames. The results on a dataset comprised of 648 AP4 echo cines demonstrated an enhancement of Dice score by an average of 3% compared to a U-net trained on the end-diastolic and end-systolic frames in a supervised manner. Figure 3 demonstrates this improvement for four sample subjects.

#### 2.1.3. Advances in Intravascular Ultrasound (IVUS) Image Segmentation and Characterization

Atherosclerosis is the build-up of plaques inside the artery walls. The rupture of atherosclerotic plaques is the major cause of acute cardiovascular events, such as cardiac infarction or stroke. Clinically, local treatment of such a rupture-prone plaque (or vulnerable plaque) in coronary arteries is percutaneous coronary intervention (PCI), which is a catheter-based procedure to open up the narrowed or blocked arteries and restore the blood flow. Thus, the detection of such vulnerable plaques is of paramount importance in clinical applications to prevent the occurrence of acute fatal events, such as heart attack and stroke and to guide PCI.

Intravascular ultrasound imaging (IVUS) is an important minimally invasive imaging technique which offers a close visualization of the coronary arteries from inside, providing a direct measurement of a few mm of the atherosclerotic plaques [78]. It is considered the gold standard for in vivo imaging of coronary arterial walls and is routinely used in clinics to assess the degree of, for instance, lumen stenosis, plaque anatomy [79]. For this purpose, segmentation of the lumen, vessel wall (intima and media layer), and plaque is required. However, the segmentation of arterial structures in IVUS images can be very challenging due to the presence of artifacts, low contrast, and poor signal-to-noise ratio. Thus, new advanced techniques for accurate segmentation are necessary.

**Figure 4:**
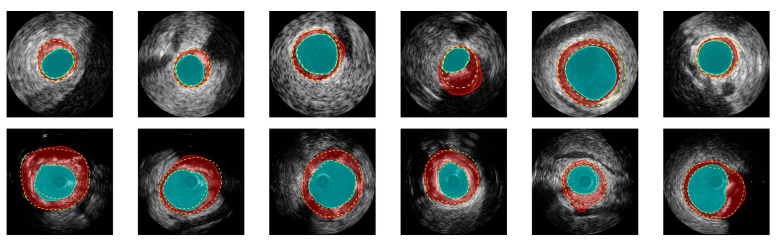
Example results of detecting the lumen and media borders for images obtained at 20 MHz (first row) and 40 MHz (second row). The segmentation results for lumen and media are shown by cyan and red colors, respectively. The yellow dashed lines show manual annotations by experts [84]. Reprint from [81] with permission.

CNNs have been widely employed on IVUS data for segmentation purposes, but large datasets are not easily acquired or available. To circumvent this problem, several groups have focused on the use of data augmentation techniques and optimizing the CNN architecture to improve the feature learning capability of the network on small datasets [80,81,82,83]. For example, in [80], the authors applied an FCN, called IVUS-Net, followed by a post-processing step on a publicly available IVUS B-mode dataset [84] to segment the lumen and media–adventitia regions of the artery. Compared with the state-of-the-art methods, their proposed method showed an improvement by 8% and 20% in terms of Hausdorff distance [85] for the lumen and the media segmentation, respectively. In a more recent study, Yang et al. [81] proposed an optimized extension of IVUS-Net, called DPU-Net, for the lumen and media–adventitia segmentation. Furthermore, to tackle the lack of training data, the authors introduced a real-time augmenter to generate more IVUS data with artifacts. The model was applied on a publicly available dataset with a center frequency of 40 MHz and 20 MHz frames, respectively [84]. The experimental results illustrated the superiority of the proposed architecture over several competing methods, such as SegNet [86] and U-net. DPU-Net also demonstrated high generalizability for predicting images in the test sets that contain a significant number of artifacts that are not presented in the training set. Figure 4 depicts a visual comparison between the manual segmentation by experts and predictions based on DPU-Net.

To further improve the performance and the generalizability of CNNs for the IVUS segmentation, Bargsten et al. [87] applied anatomical constraints to train a U-net architecture. These constraints were represented by regularization terms which considered some prior knowledge about the lumen and vessel wall, such as location and shape. Compared to a baseline U-net model, the experimental results showed a performance improvement of up to 59.3% in terms of the modified Hausdorff distance.

In addition to the lumen and vessel wall segmentation, several other studies in the field employed CNN-based models for plaque segmentation. These studies usually use a two-stage segmentation framework: a network for plaque region localization followed by a segmentation network. For example, Olender et al. [88] used a CNN architecture for arterial tissue classification. The method comprised three steps. First, the area between the lumen-intima border and the media–adventitia border were identified. This region was then divided into pathological and non-pathological tissue. Pathological areas were then fed into a CNN architecture for plaque-type classification. The experimental results showed an overall accuracy of 93.5%. Li et al. [89] presented a U-net architecture in a two-stage pipeline to segment calcified plaque, luminal regions, and media–adventitia. In the first stage, a U-net architecture segmented the lumen and media–adventitia regions. Then, the output of this stage was provided to another U-net architecture for the calcified plaque identification. Using a two-stage U-net prevented the model from recognizing bright speckle-noise outside the plaque as the calcification. The proposed model was applied on a dataset containing 713 grayscale IVUS images with three different loss functions. The proposed method showed high accuracy even when the target vessel was surrounded by shadow artifacts or side vessels.

#### 2.1.4. Advances of Point of Care Ultrasound (POCUS)

Point of care ultrasound (POCUS) refers to ultrasound examination outside the ultrasound lab, such as bedside care, ambulant care, or in emergency departments. POCUS has been a widely used tool for imaging and therefore reducing the time in clinical decision-making ([90]), pediatric emergency, medical education. It has achieved even more success because of the development of portable technologies as well as increased availability of POCUS machines [91,92,93]. There are still barriers to widespread use of POCUS because of the lack of a structured curriculum to educate physicians [94].

Kimura [95] presented a review of literature for point of care cardiac ultrasound techniques for physical examination. It provides insight on the utility of POCUS in detection of left atrial enlargement, signs of left ventricular systolic dysfunction, lung congestion, and elevated central venous pressures which are missed in the routine cardiac examination. It also focused on the utility of POCUS as a standard physical examination in cardiovascular medicine for augmenting cardiac physical examination and improving bedside diagnosis. These devices play a very important role in screening, complementing the abilities of physicians for performing cardiac auscultation [96]. The importance of handheld echocardiography has been studied extensively and it was shown that pocket size echocardiography (PSE) combined with other tests had a significant impact on the cardiology examination helping in finding the proper diagnosis [97]. Additionally, the benefits of the devices can be increased if proper training of personnel is done so that they can use these devices correctly, and with ease. Fox et al. [98] studied the impact of student volunteers with minimal training on the screening of Hypertrophic Cardiomyopathy (HCM) which is a life-threatening condition. The number of participants involved were 2332, and it was found that the volunteers were able to successfully screen for HCM with a sensitivity of 100%.

Kalagara et al. [99] in their review discussed the utility of POCUS for various clinical tasks such as in the operating room (OR), preoperative clinic, intensive care unit (ICU) and concluded that it is a valuable diagnostic bedside tool. They also discussed the affordability of the ultrasound systems, POCUS related education as well as the benefits of the POCUS in the clinical side. Gaspari et al. [100] performed a study based on 20 hospitals (793 patients) including patients from Advanced Cardiac Life Support (ACLS). Ultrasound was performed before and after the ACLS and it was found that the POCUS of the cardiac activity was the most important variable for deciding survival to hospital admission, survival to hospital discharge and return of spontaneous circulation. There have been many efforts to discuss these approaches and the common limitations of these techniques. Since these approaches are becoming quite popular the need to educate the practitioners for acquiring high-quality images, and interpreting, is becoming increasingly urgent [101].

The use of DL-based methods for POCUS imaging is a rapidly developing field. A review of the popular and most recent architectures was done by Blaivas and Blaivas [102] using AlexNet, VGG-16, VGG-19, ResNet50, DenseNet201, and Inception-v4. They used a public dataset with 750,018 individual ultrasound images of five different types and showed that the classification accuracy varied from 96% to 85.6% for the various models, with VGG-16 giving the best performance while the DenseNet201 performed the worst for classification. Another work by Blaivas et al. [103] proposed a LSTM network for inferior vena cava (IVC) POCUS videos in patients undergoing the intravenous fluid resuscitation and use 211 videos and achieved the receiver operating characteristic curve of 0.70 (95% confidence interval [CI], 0.43–1.00) for predicting the fluid responsiveness. Generative Adversarial Networks (GANS) have also gained popularity for generating more data as well as applicable in the cases where the paired input/output pairs are not easily available for training the models. Using the idea, Khan et al. [104] proposed a CycleGAN for improving the contrast and resolution of POCUS images for images acquired in vivo as well as phantoms. Thus, recently DL-based models have gained a lot of importance in the advanced development of POCUS-based imaging.

Another research area where DL is making significant progress is in improving the quality of image acquisition using POCUS [105]. Blaivas et al. [106] developed a DL-based model for image quality assurance for automatic image classification. They used a large dataset of 121,000 images extracted from US sequences and had an accuracy of 98%. Cheema et al. [107] highlighted the importance of DL-based models trained on highly skilled cardiac sonographers to train novice users to acquire high-quality images which can be easily extended to POCUS systems. Shokoohi et al. [105] further emphasized on using DL-based models for removing the background noise, which can help in training newly trained sonographers by focusing them on finding specific features and hence enhancing the image quality. Thus, DL-based models are also helpful in acquiring good quality images in POCUS-based systems.

In summary, we have outlined all the aforementioned applications of major DL-based models in Table 1.

## 3. PA Imaging and DL Techniques in Cardiology

### 3.1. The Development of PA Imaging Techniques in Cardiology

The detection of the vulnerable plaque is crucial to guide cardiovascular interventions and thus prevent the occurrences of the acute cardiac events. The vulnerability of the plaques is highly related to their compositions. Specifically, the typical composition of the vulnerable plaques can be concluded as the presence of lipid, calcification, intraplaque hemorrhage and macrophages [108,109]. All these typical components in vulnerable plaques can be well visualized by PA imaging, making PA imaging a very powerful tool to characterize vulnerable plaques. Over recent years, PA imaging for vulnerable plaque detection and characterization has become a massive research topic with a lot of ongoing efforts.

In general, there are two typical approaches in PA imaging of vulnerable plaques: endoscopic catheter-based PA imaging, i.e., intravascular PA (IVPA) imaging, and non-invasive PA imaging. In the following section, the major technological developments of both PA imaging approaches are reviewed.

### 3.2. Intravascular PA Imaging of Vulnerable Atherosclerotic Plaques

#### 3.2.1. IVPA Imaging Catheter Development

**Figure 5:**
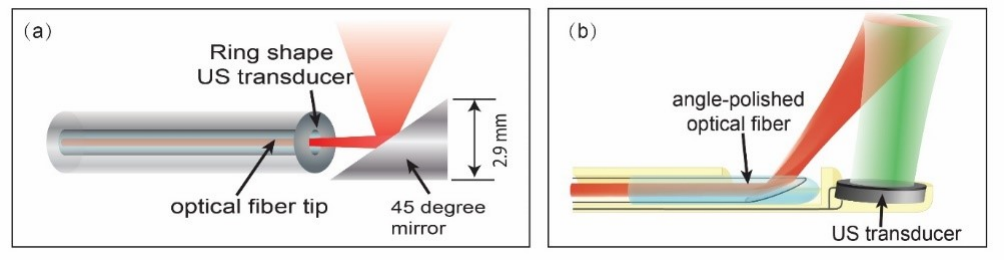
Schematic of different IVPA catheter designs. (**a**) Schematic of a collinear IVPA catheter design. (**b**) Schematic of an IVPA catheter with a longitudinal offset between optical and acoustic beams (red optical beam and green ultrasound beam). Reprinted from [114] with permission.

As an essential part of the general IVPA imaging system, an IVPA catheter mainly consists of a light delivery part, and an ultrasound transducer. A good IVPA catheter requires small dimensions, high imaging sensitivity, and sufficient mechanical support while advancing in the coronary arteries. It is one of the key challenges for the application of IVPA imaging to detect vulnerable plaques. So far, there are two typical designs of a IVPA catheter based on the configuration of light delivery and an US transducer: a co-linear design and an offset design, which are shown in Figure 5. The co-linear design offers the most overlap between the optical and acoustic beams, resulting in a higher imaging sensitivity; however, miniaturization is difficult. Cao. et al. developed the first co-linear IVPA catheter with the outer diameter of 1.6 mm [110]. The second catheter design, with an offset (longitudinally or laterally) between the optical and acoustic beams, is preferred in practice due to its great potential of miniaturization. However, the offset in the catheter can lead to signal loss when the imaging targets are close by and far away from the transducer [111,112]. The smallest IVPA catheter reported so far has a diameter of 0.09 mm [113].

#### 3.2.2. IVPA Imaging of Diverse Compositions in Vulnerable Plaques

**Figure 6:**
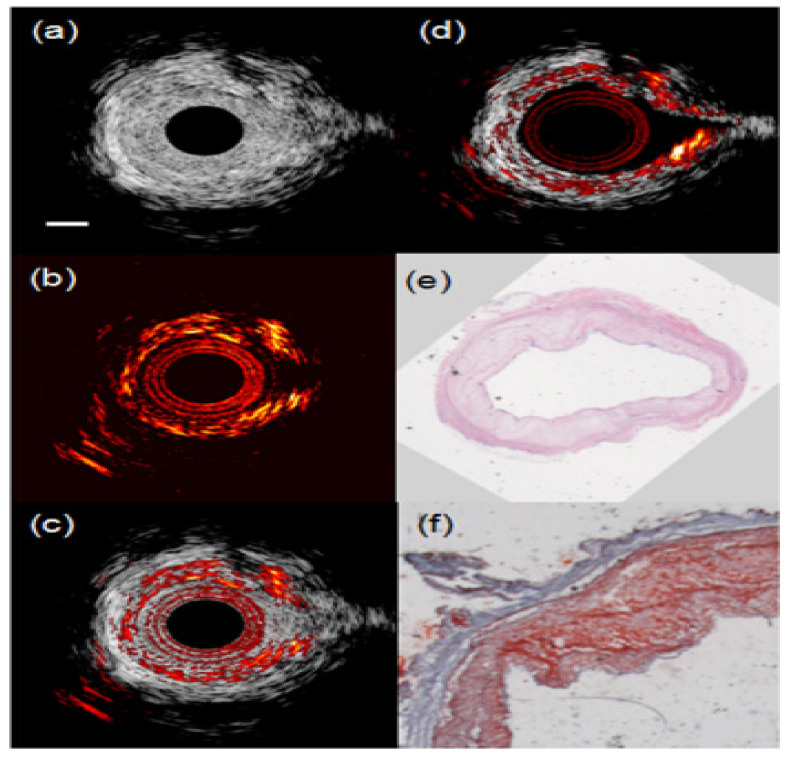
(**a**) IVUS, (**b**) IVPA, and (**c**) combined IVUS/IVPA images of an atherosclerotic rabbit aorta acquired in the presence of blood. (**d**) Combined IVUS/IVPA image of the same cross section of the aorta imaged in saline. IVUS and IVPA images are displayed at 35 dB and 20 dB, respectively. The scale bar is 1 mm. (**e**) H&E and (**f**) Oil red O stain of the tissue slice adjacent to the imaged tissue cross section indicate that the aorta has lipid-rich plaque. (Reprint from [122] with permission).

As mentioned before, compositions such as lipid accumulations, intraplaque hemorrhages, and inflammation can be imaged and are used as effective indicators to detect vulnerable plaques with IVPA imaging. Among these compositions, lipid is the most commonly used PA biomarker and has been studied intensively [9,110,115,116,117,118,119,120,121]. It is well established that the best wavelengths for imaging lipid-rich plaque is around 1200 nm and 1700 nm [116]. It is even possible to image lipid in the presence of blood [122]. Figure 6 shows an IVPA image of a lipid-rich plaque in a rabbit aorta through blood.

**Figure 7:**
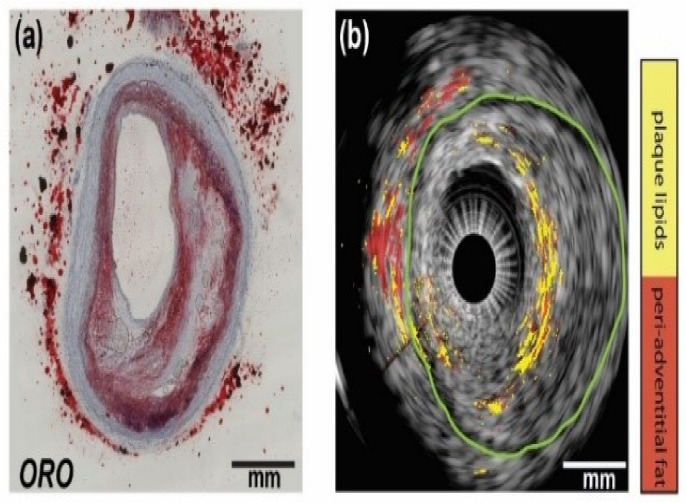
Ex vivo lipid differentiation result of an atherosclerotic human coronary artery. (**a**) Histology: Oil Red O staining of the IVPA/IVUS imaging cross section (lipids are in red). (**b**) Lipid differentiation map overlaid on a co-registered US image of the coronary artery. The lipids in plaques are in yellow whereas lipids in the peri-adventitial tissue are in red. The dynamic range of the US image is 45 dB. Reprint from [123] with permission.

Moreover, multispectral PA imaging has been proposed to characterize different lipid types in a plaque as well as the surrounding peri-adventitial adipose tissue with only two wavelengths (Figure 7) [123]. A further characterization of the lipid’s PA spectral signatures in human plaques (and) corresponding molecular validation has been achieved recently based on a novel PA slide microscope (μsPA) system [124]. As lipids are involved in all stages of the development of plaques, a comprehensive characterization of lipids can potentially guide the development of PA-based atherosclerosis disease staging [124].

As another key component involved in the pathology of atherosclerosis, macrophages are present at a relatively early stage in atherosclerosis due to the initial inflammation in the arterial endothelial layer. Macrophages can accelerate the progression of atherosclerosis by the release of matrix metalloproteinases (MMPs), which weaken the fibrous cap and make the plaques more prone to rupture. Therefore, the visualization of macrophages or MMPs can detect vulnerable atherosclerotic plaques at an early stage. However, due to their insufficient endogenous PA contrast, it requires special PA contrast agents to visualize macrophages and MMPs.

**Figure 8:**
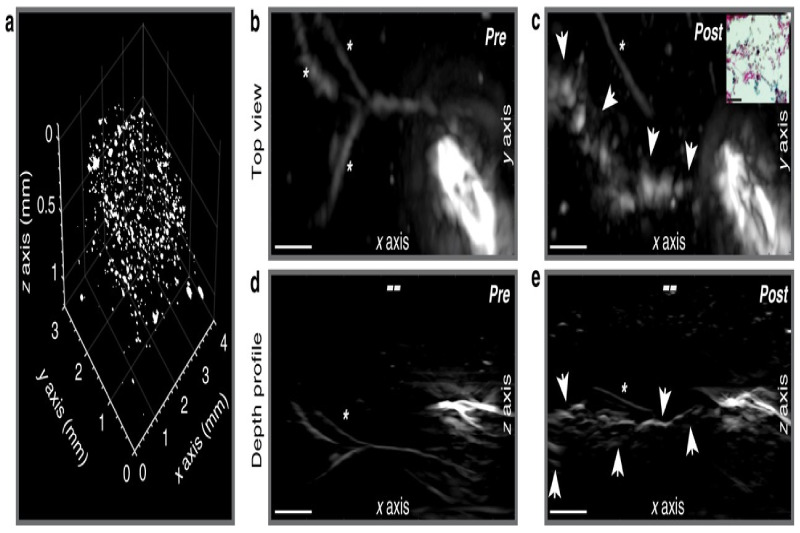
HDP facilitates single-cell visualization with raster-scan optoacoustic mesoscopy (RSOM). Signals of HDP-laden primary macrophages are separated from hemoglobin in blood-agar phantoms and depicted in a volumetric scatter plot. Subcutaneous injection in the dorsal area of a FoxN1 nude mouse of the cells measured in (**a**). A catheter was used to determine the injection area and scans were recorded pre- (**b**,**d**) and post (**c**,**e**) cell injection showing the top view and a depth profile. The opening of the needle is seen on the right side of the images from which the macrophages emerge post injection as a dense line-up (arrows), 0.7–1 mm below the skin surface (–). Blood vessels are faintly detected at 630 nm and indicated by *. Scale bars are 500 µm in x, y, and z. Inset in panel (**c**) shows labeled macrophages in histological tissue sections with Schmorl’s staining. The outtake corresponds to an area near the needle tip. Scale bar is 50 µm. Reprinted from [129] with permission.

Contrast agents such as gold nanoparticles and organic dyes such as ICG or ICG-based PA nanoprobes were introduced to selectively label the macrophages and MMPs, and enhance the PA visualization [125,126,127,128]. Later, Weidenfeld et al. introduced a novel homogentisic acid-derived pigment (HDP) as a biocompatible label to “paint macrophages black”, which can be easily visualized by PA imaging [129]. The PA image of such HDP-labeled macrophages is shown in Figure 8. This HDP cell label has the great potential for in vivo applications and will provide new insights into the behavior of macrophages during different pathophysiological states of atherosclerosis.

#### 3.2.3. Towards In Vivo IVPA Imaging of Vulnerable Atherosclerotic Plaques

To move towards in vivo clinical applications, ongoing efforts to develop a real-time IVPA imaging system and to initialize in vivo PA imaging in animal models were made. Wu et al. developed a real-time IVPA/US imaging system capable of IVPA imaging of lipid-rich plaques in a swine model at 20 frames per second in vivo [9]. Later, Xie et al. developed a new IVPA imaging system that can reach an imaging speed as fast as 100 frames per second and can imaging without blood flush [130]. All these results showcase the great potential of clinical translation of IVPA imaging to detect vulnerable plaques and therefore guide PCI.

### 3.3. Non-Invasive PA Imaging for Cardiovascular Applications

As PA imaging is very sensitive to different types of hemoglobin, it can be a non-invasive and cost-effective imaging method for the detection of vulnerable plaques with intraplaque hemorrhages and for extra cardiovascular hemodynamic measurement (such as blood flow and oxygen saturation, etc.) to facilitate accurate diagnosis and prevention of CVDs.

**Figure 9:**
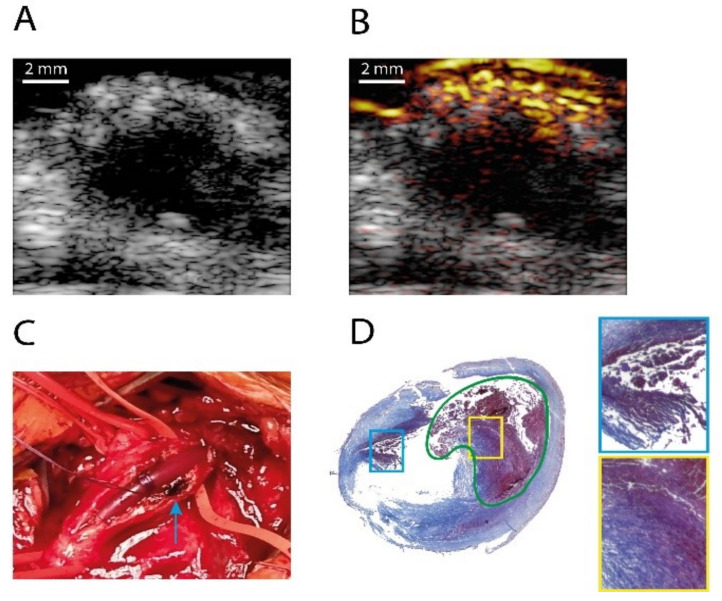
In vivo PA and US image of a human carotid artery with intraplaque hemorrhage; (**A**) US image; (**B**) overlaid PA/US image (808 nm, dynamic range 23 dB); (**C**) photo of the carotid plaque during the CEA surgery; (**D**) Masson’s trichrome staining of the artery. The area indicated in green is a lipid core filled with a large hemorrhage. The highlighted boxes show two regions of hemorrhages found in the plaque. Reprinted from [132] with permission.

Arabul et al. presented the first PA images of intraplaque hemorrhages from human carotid plaques based on a diode-based handheld PA imaging system with limited optical wavelengths (one or two) [131]. Recently, with the updated version of the PA imaging system, Muller et al. reported the first in vivo clinical results, i.e., intra-operative PA imaging of intraplaque hemorrhages in carotid artery plaques [132]. This unique intra-operative study can facilitate a more comprehensive understanding of the properties of the PA signals generated from intraplaque hemorrhages. In this study, strong PA response were related to the presence of the intraplaque hemorrhages (Figure 9), and a diffused signal pattern was observed in the hemorrhage lesion, probably caused by the heterogeneity in the composition of the plaque [132].

**Figure 10:**
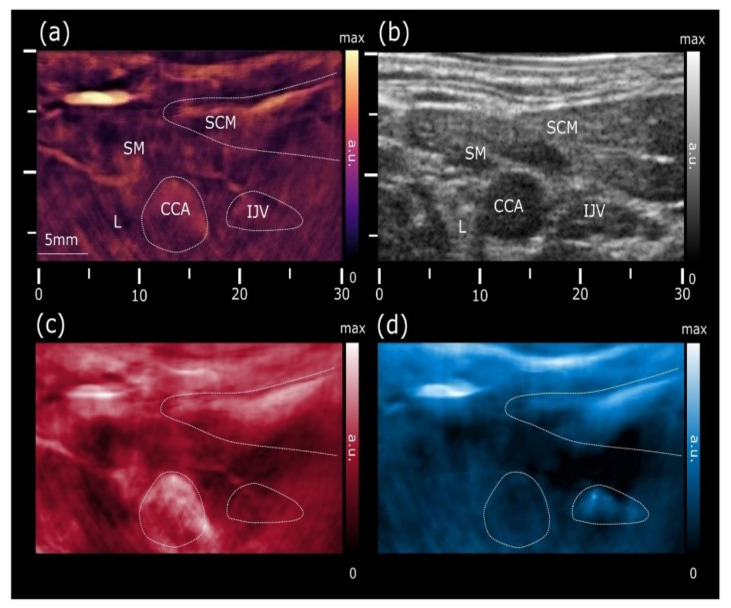
PA image of the common carotid artery based on the MSOT system. (**a**) PA image at 800 nm shows increased vascularization of the skin, strap and sternocleidomastoid muscles, allowing for a clear identification of the common carotid artery and internal jugular vein. (**b**) US image revealing the common carotid artery and jugular vein as echo-free structures. (**c**) Map of the unmixed distribution of oxygenated hemoglobin (HbO2). (**d**) The corresponding map of the deoxygenated hemoglobin (Hb). CCA: common carotid artery; STM: sternocleidomastoid muscle; SM: strap muscle; IJV: internal jugular vein; L: thyroid lobe. Reprinted from [134] with permission.

Another advanced and handheld-based multispectral optoacoustic tomography system (MSOT) was developed and implemented by the research group from the Technical University of Munich, Germany. The MSOT system typically uses a single-pulse-per-frame (SPPF) acquisition scheme to minimize motion artifacts, and it typically operates in the “optical window” of 680–980 nm for a deeper imaging depth for soft biomedical tissues [133]. The MSOT system has been applied in various CVD applications in vivo both in animal and in human [134,135,136,137,138,139]. Figure 10 is an example of non-invasive PA imaging of the carotid artery to estimate the oxygenation in vivo. Please note that the MSOT systems have been given clinical approval, which may enable more opportunities of (pre)clinical studies for a wide range of diagnostic imaging applications in general. Specifically, promising results have been reported recently and demonstrated the great potential of MSOT to visualize vulnerable plaque in carotid artery in patient [140,141], which may accelerate the clinical translation of PA imaging in cardiology.

Another study by Kang [142,143] introduced a new concept of a non-invasive PA-based indicator dilution measurement, and developed an advanced method to measure the cardiac output, which is an important hemodynamic parameter for assessment of cardiac function, and is especially helpful for monitoring and optimizing the fluid status in high-risk surgical and critically ill patients.

### 3.4. PA Imaging of Cardiac Arrhythmia

Atrial fibrillation (AF) is a common and persistent cardiac arrhythmia with high morbidity and mortality rates [144] and is associated with a high risk of stroke and heart failure. Currently, catheter-based radiofrequency (RF) ablation to interrupt the aberrant conduction paths in the heart is an effective treatment of AF. However, many complications such as the control of the catheter and pulmonary vein reconnection are typically present during the RF ablation, making it a long-lasting and low success rate procedure (the success rate is generally 60–80% even including secondary ablations). To overcome the challenges related to ablation, accurate real-time feedback on the lesion formation during ablation, as well as post-treatment lesion assessment is necessary.

**Figure 11:**
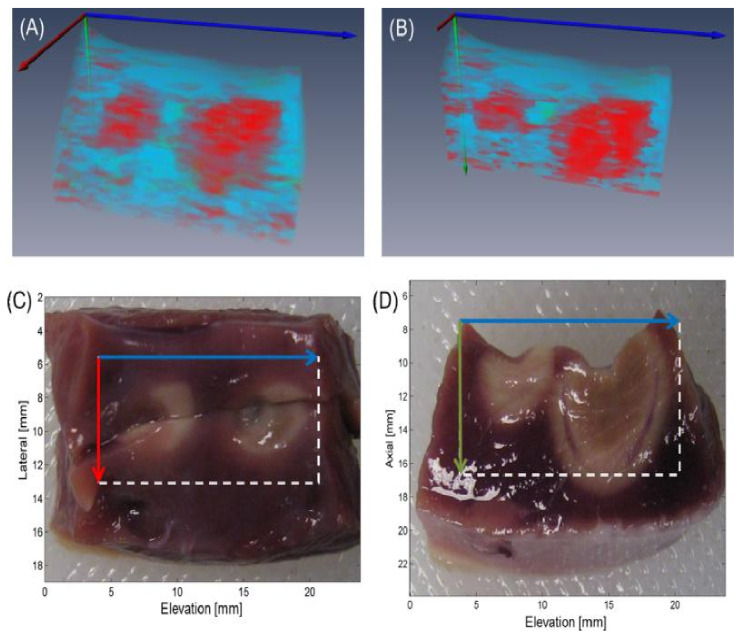
Three-dimensional rendering (**A**) of TCM volume with clipping plane corresponding to tissue bisection (**B**). Matching top- (**C**) and side-view (**D**) gross pathology photographs with axes and FOVs indicated by arrows and boxes, respectively. Reprinted from [147] with permission.

Multispectral photoacoustic imaging is powerful for tissue characterization, and many studies have explored the possibility of multispectral photoacoustic imaging to visualize the underlying structures and lesion gaps during RF ablation [121,145,146,147,148], showing very promising results. Figure 11 is an example of PA -based differentiation between the ablated and non-ablated regions. It was found that PA spectral differences were clearly observed between non-ablated and ablated regions, and that these spectral differences can be related to changes in the hemichrome, methmyoglobin, and protein denaturalization content of the tissue [146].

**Figure 12:**
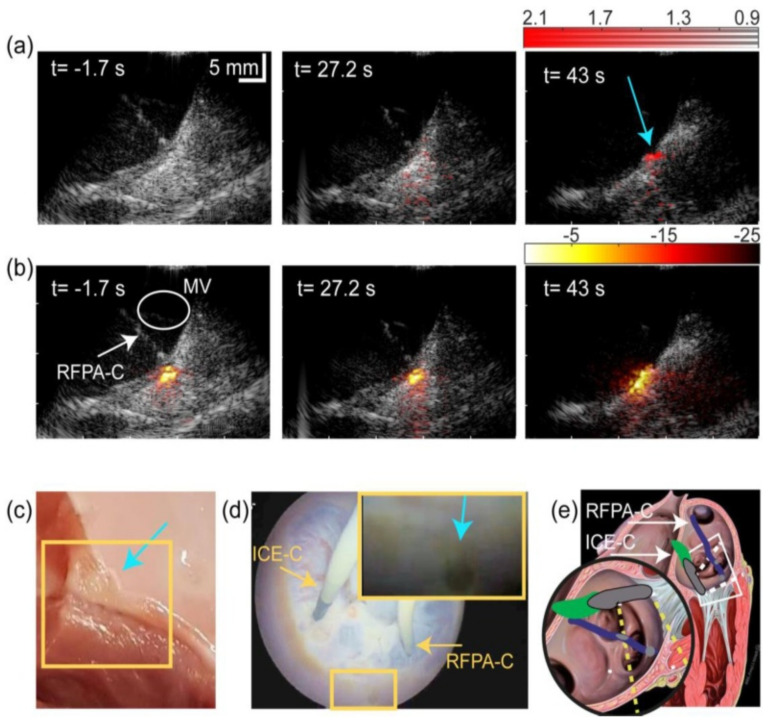
Ablation monitoring in a beating heart. (**a**) 2λPA images before, during and after ablation, available as Movie 2. (**b**) I790 equivalents. 2λPA data confirm lesion formation. (**c**) Photograph of lesion made. (**d**) Video endoscopy frame confirming a lesion was made. (**e**) Sketch of instruments positions. Round inset: ICE-C and RFPA-C relative to the valve, oriented as in the images in (**a**,**b**). ICE catheter (ICE-C); PA-enabled ablation catheter (RFPA-C). Mitral valve (MV). Cyan arrows indicate indentation formed by ablation. Reprinted from [146] with permission.

To move towards the clinical application of PA imaging guided RF ablation, Iskander-Rikz introduced a new design of intracardiac ablation imaging, and explored the possibility of two wavelength (790 nm and 930 nm) PA imaging to characterize ablation, and successfully validated the method ex vivo. The results shown in Figure 12 demonstrated that the dual wavelength photoacoustics can provide real-time monitoring of intra-atrial RF ablation procedures in a blood-filled beating heart. Real-time visualization of ablation lesion formation and lesion gaps was achieved with a modified clinical device consisting of a custom ablation catheter (modified for illumination) and intracardiac echography (ICE) for signal acquisition. This setup provides a good solution for the clinical translation of PA imaging to guide RF ablation. Another study from Li et al. [149] proposed a new strategy to enhance the internal illumination based on the designed graded-scattering fiber diffuser, which may be applied to improve the optical illumination for PA imaging of ablation progression.

Moreover, a new study by Ozsoy et al. [150] recently proposed a sparse PA sensing (SOS) technique for ultrafast four-dimensional imaging of cardiac mechanical wave propagation. This dedicated system can characterize the cardiac mechanical waves at high contrast, high spatial resolution (around 115µm) and sub-millisecond temporal resolution in murine models, which can further enhance the understanding of the cardiac function in arrhythmia.

### 3.5. Application of DL in PA Imaging in Cardiology

Although PA imaging is still a relatively new imaging modality and is at an early phase along its revolution path, increased attention is devoted to DL techniques in the PA imaging field, and the relevant studies are booming, especially in the last few years. However, unlike US imaging, which has been widely applied in clinics in cardiology, PA imaging is still at the pre-clinical phase so far. Moreover, DL techniques have not been spread in PA imaging for cardiology as largely as in the case of US imaging. There are many recent studies to comprehensively review the applications of DL in PA imaging in general [151,152,153]. Here, in this section, we only briefly introduce the DL-based applications related to PA imaging in cardiology, which can be simply summarized as the application of DL in PA image reconstruction, PA imaging quantification, and tissue segmentation [151].

Among the three applications mentioned above, DL-based PA image reconstruction is the most popular topic [17,18,154,155,156,157,158,159]. Due to the broad-band nature of PA signal and non-ideal data acquisition, the conventional PA image reconstruction method, such as delay and sum, usually results in the degradation of image quality due to information loss and high artifacts and noise. DL-based image reconstruction, which can be mainly summarized into the learning-based post-processing reconstruction and the model-based learning reconstruction methods, can reduce the artifacts and background noise in PA images and then improve overall imaging quality [157]. A recent study from Lan et al. [159] demonstrated the application of DL in PA imaging reconstruction for in vivo imaging of the human palm with great success.

Moreover, DL techniques also play an essential role in PA quantification imaging. For instance, DL can help to estimate oxygenation saturation, which is an important physiological parameter to assess metabolic function in clinics. Cai et al. [160] employed a ResU-net (a U-net with residual blocks) on 2D multi-wavelength PA images to estimate the oxygen saturation and the absolute concentration of indocyanine green. The experimental results demonstrated the high accuracy of the proposed method and its robustness to the optical property variations. Moreover, DL techniques have also been applied for automated segmentation of vascular structure in PA images [161,162]. Chlis et al. [161] used a sparse U-net model to identify the most important illumination wavelengths while segmenting the blood vessels (arteries and veins) in clinical multispectral PA (MSOT) images. The experimental results on a dataset with 33 images showed a performance comparable with a standard U-net. More recently, the study from Gröhl et al. [163] has demonstrated the feasibility of using DL for fully automatic multi-label tissue annotation in multispectral PA images in humans. The combination of these DL-based vascular segmentation and oxygen saturation measurements could potentially be useful for assessing cardiac functions in clinics.

## 4. Discussion and Future Opportunities

Since the first application of US imaging in cardiology, we have witnessed many advancements in US imaging, which has been widely used in clinics to diagnose various CVDs. In recent years, with the introduction of DL techniques, which can provide good performance as well as fast and real-time solution, learning-based advanced US imaging has gained considerable attention for different cardiology applications. In this paper, we reviewed some typical work of these learning-based US image analysis methods ranging from selecting a view, performing the required segmentation, and finally, the application in point of care ultrasound imaging. We discussed some of the most effective DL-based segmentation methods on US images. Current learning-based US segmentation methods are mainly based on CNN models. Some research studies focused on improving the feature learning capabilities of CNNs by optimizing the network architecture and including shape constraint-based loss. Others used a hybrid framework by combining CNNs with other DL or traditional machine learning methods to include additional information, such as temporal dependency between consecutive US slices, to further enhance US cardiac image segmentation performance. However, based on current results from the literature, more efforts are required to translate these segmentation methods to clinical practice. DL-based segmentation methods require large and high-quality annotated datasets to perform and generalize well. This requirement, however, has been rarely satisfied, especially in the field of medical imaging, where data collection and annotating are challenging and expensive procedures. To tackle this problem, data augmentation techniques have been commonly used. Effective data augmentation, however, needs domain knowledge. Furthermore, augmented data might not necessarily present all possible variants of clinical data. Thus, developing task-specific augmentation methods from existing data using generative models such as GANs and adversarial example generation is crucial and needs to be more investigated in future research.

Another area where DL-based models are making an impact is POCUS imaging. POCUS imaging would also be an important trend in future clinical applications due to its great flexibility. The development of cost-effective and easily integrable hardware combined with lightweight networks will also benefit POCUS imaging.

Contrary to US imaging, PA imaging is currently still in the research and pre-clinical phase. However, due to its hybrid nature, PA imaging could be a perfect imaging modality next to US imaging and can provide complementary information such as tissue compositions. These features make PA imaging especially useful for the characterization of vulnerable plaques in cardiology. As reviewed in this paper, research efforts are ongoing to move PA imaging forward along its clinical translation path. For instance, a Dutch start-up company has further developed the IVPA techniques for potential eventual use in patients since 2020. Moreover, various studies have been done using DL to improve PA image reconstruction and image processing tasks. The application of DL techniques to improve the PA reconstruction based on the co-registered US information as in the study proposed by yang et al. [164] would be interesting to explore in the future. Despite a lot of ongoing efforts, the application of DL on PA data in CVD is not mature enough. The efforts have been limited to several studies on blood vessel segmentation [161] and estimating the oxygenation saturation so far. The major challenge that restricts the application of DL on PA data is the lack of high-quality labeled experimental data. To tackle this issue, most research studies have mainly focused on using simulated data for training DL models, but it leads to a drop in performance when tested on the experimental data due to the different data distribution used in the training and inference phases. Domain adaptation methods [165,166] could help in reducing the gap between the distribution of simulated data and real-PA data.

Recent studies have established that atherosclerotic plaque composition is a crucial and informative factor for identifying patients at risk of fatal cardiovascular events [3]. IVUS has been recently used for the identification of calcified plaque-type [88]. However, it is not a suitable imaging modality for the characterization of all plaque components. In contrast, PA imaging is considered to be a promising modality for identifying plaque components using multiple wavelengths, and, to this end, and many different PA spectral unmixing techniques have been developed [167,168,169,170]. To further improve the capability of PA characterization of plaque compositions, more effort should be put in the direction of application of DL techniques for plaque decomposition in PA images acquired from human plaque lesions.

In general, the current state-of-the-art DL methods for CVD applications consider pixel-value information of images to diagnose and assess the disease. However, in practice, accurate non-imaging data based on the clinical records enable cardiologists to interpret imaging findings appropriately, leading to more accurate diagnosis, disease assessment, and decision-making. Thus, the integration of imaging data with clinical records needs to be more studied in the context of DL.

Another key aspect is that most published studies for DL in cardiovascular US/PA imaging are in the context of exploratory and preliminary applications. Thus, they suffer from the lack of validation on the large cohort, multi-center datasets. Therefore, there is no guarantee of the generalization performance of these studies. To better diagnose CVDs, a multi-modality imaging method combined with DL techniques would be a good future option. For instance, the combination of IVUS/IVPA and cardiac US imaging may allow both a global and local visualization of cardiovascular lesions. However, the registration between different imaging modalities at different length scales, imaging positions, and time frames is required, and these challenging image registration problems may be solved with the help of the data-driven DL methods.

## Figures and Tables

**Table 1:** Popular DL models used for various cardiac ultrasound applications.

Application	Popular Deep Learning Models
Cardiac viewpoint classification	Custom architecture based on VGG, ResNet, DenseNet [50]; Custom architecture based on CNNs [49]; Custom architecture fusing spatial and temporal information using CNNs [48]
LV segmentation	U-net-based architectures [59,60,62,63,71]; CNN [61]; Deep belief network (DBN) [55,68,69,70,74]; U-net combined with RNNs [64,67,75]; U-net with TL-net [72,77]
IVUS image segmentation	U-net-based architectures [80,81,83,87,89], Autoencoder [82], CNN [88]
Point of care ultrasound (POCUS)	AlexNet, VGG-16, VGG-19, ResNet50, DenseNet201 [102]; LSTM [103]; CycleGAN [104];

## Data Availability

Not applicable.
